# Bibliographical

**Published:** 1856-11

**Authors:** 


### BIBLIOGRAPHICAL.

We have had upon our table for some time, "The Causes and Curative Treatment of Sterility, with a Preliminary statement of the Physiology of Generation, with colored Lithographs and numerous wood cut illustrations, by Augustus K. Gardner, A. M., M. D., &c., &c.," published by De Witt & Davenport, 160 and 162 Naussau Street, New York, which doubtless contains much useful, if not *new* matter, and upon the whole, may be looked upon as rather a valuable book, though decidedly marred by looseness of style, and what we are inclined to consider, as a touch of obscenity.

The most objectionable feature, however, connected with the publication, is the minute detail of the contents of the work, in an advertisement in the New York Tribune (an extract from which we find in the Western Lancet) intended, of course, for the public eye, which we do not hesitate to pronounce not only decidedly quackish, but indecent; and for which we consider that the author should be held responsible; the sort of notoriety acquired for him by the character of the work, and the course pursued by his publishers, can hardly, we think, be considered very creditable.

"Clinical Lectures on the Diseases of Women and Children, by Gunning S. Bedford, A. M., M. D., Professor of Obstetrics, the Diseases of Women and Children and Clinical Midwifery in the University of New York." Published by Samuel S. & Wm. Wood, 389 Broadway, New York. We have to return our thanks to the author for the *fourth* edition of the above work, the *first* appearance of which was only some sixteen months ago—upon the reception of the second edition, we expressed our high appreciation of the value of the work, and take great pleasure in commending this enlarged and revised edition of September, 1856. But few medical works have ever been so enthusiastically received as this of Prof. Bedford's.

"Human Physiology, Statical and Dynamical, or The Conditions and Course of the Life of Man, by John William Draper, M. D. L. L. D., Professor of Chemistry an Physiology in the University of New York, illustrated with nearly three hundred wood engravings, New York. Harper & Brothers Publishers, Franklin Square, 1856."

As acknowledged in the last number of our Journal, the above work has been received from the publishers, for which we can very sincerely return our thanks, not only for the courtesy, but for the valuable book thus put in our possession. We have been, for some time, expecting a work from this source, and are truly gratified that Prof. Draper has furnished us with an *American* work on Physiology, that will compare favorably with the production of any foreign author; even with the great work on this science, the "Principles of Human Physiology," by Carpenter.

The work before us professes to be the text of the Lectures on Physiology, which the author has given in the University of New York, and is divided into two branches, Statical and Dynamical Physiology, in relation to which he remarks that, "The expediency of this has been impressed upon his attention, by the necessity of conforming his course of lectures to the wants of a medical class; the physician is chiefly concerned with the conditions of life—the organic functions, as digestion, respiration, secretion, &c. The doctrines of development and the career of an organic form, are of less pressing interest. But it was very soon found that other advantages were derived from this subdivision, as might indeed have been expected, from its conformity to the usages of writers on other branches of physical science, doubtless if such a separation be accepted by physiological authorities, it will tend to the more rapid progress of both portions of the subject, by imparting to each a more definite office."

The object of the author seems to have been to make a practical work, and to aid in the removal of the mysticism which has pervaded the science, and remarks, "Alone, of all the great departments of knowledge, Physiology still retains the metaphysical conceptions of the Middle Ages, from which Astronomy and Chemistry have made themselves free. To exorcise it from such nonentities as irritability, plastic power, vital force, is the duty of the rising generation of physicians—it is also their interest. Empiricism will never be banished from the practice of medicine until Physiology is made an exact science."

We fully concur with him in the view presented, that "to the medical profession, as matters now stand, nothing is of more importance than the dissemination of physiological knowledge. Empiricism could not flourish as it does if the structure and functions of the body of man were better understood. How many advantages would arise if the elements of this science were made a part of general education in America. What branch of knowledge has intrinsically a better title thereto? Is it all to be wondered at, that every kind of medical imposture prospers in communities where almost every one believes that a man has one rib less than a woman, and even among persons pretending to education, scarcely one can be found who has a distinct idea of the size, shape and position of his own stomach?

Such a diffusion of physiological knoweldge would not only tend to a repression of empiricism, but would also exert an effect in raising the standard of acquirement among medical men themselves. That a great revolution is impending in the practice of medicine, no one who is at all observant of the progress of science can doubt, the great physicians of the future will be the great physiologists. He who can best correct the imperfections of a machine, is he who best knows its structure and action.

Why is it, that from Astronomy, Chemistry, Mechanical

Engineering and such other subjects, empiricism has disappeared? Is it not because exact knowledge has taken the place of speculation and mysticism? The delusions of Astrology, Alchemy and Magic have been unable to maintain themselves against simple Truth. And so of the numerous medical impostures of our times, they will die out as an exact knowledge of the structure and functions of man prevails."

Without attempting any thing like a minute analysis of the work, which would, indeed, be a task of great labor, we have no hesitation in saying, that this is one of the most important additions that we have had to the list of American medical works.

We have also received "The History and Statistics of Ovariotomy and the circumstances under which the operation may be regarded as safe and expedient, being a Dissertation to which the prize of the Massachusetts Medical Society was awarded, May, 1856, by George H. Lyman, M. D.;" containing a synopsis and analysis of three hundred cases of operation for removal of diseased ovaria; some items of which we present. Of the three hundred cases, the operation was completed by the removal of the tumor in 208—the tumor could not be removed in 78—the tumor was partially removed in 10—the removal of the tumor is not mentioned in 4. In one case the result is not stated—of the remaining 299 operations 179 recovered, 120 died. Of the 208 cases in which the operation was completed, 118 recovered and 89 died. We have, then, 300 operations (we presume well authenticated) for the removal of ovarian disease, of which 119 were successful in the removal of the disease and the recovery of the patient.

In conclusion, we would commend it as a valuable monograph upon this subject; it contains 146 pages, and is printed, by John Wilson & Son, 22 School street, Boston, through whom, we presume, it could be obtianed.

